# Prophages and their interactions with lytic phages in the human gut microbiota and their impact on microbial diversity, gut health, and disease

**DOI:** 10.1128/aem.01899-25

**Published:** 2025-11-24

**Authors:** Song Zhang, Maheswaran Easwaran, Mahmoud Elafify, Aminu Abdullahi Mahmoud, Xiaoyu Wang, Juhee Ahn

**Affiliations:** 1 Future Food Laboratory, Innovation Center of Yangtze River Delta, Zhejiang University 723177 , Jiaxing, Zhejiang, China; 2 Centre for Biosciences and Biotechnology, Department of Research Analytics, Saveetha Dental College and Hospitals, Saveetha Institute of Medical and Technical Sciences, Saveetha University 194347, Chennai, India; 3 College of Biosystems Engineering and Food Science, Zhejiang University 366093 , Hangzhou, Zhejiang, China; 4 Department of Food Hygiene and Control, Faculty of Veterinary Medicine, Mansoura University 158400, Mansoura, Egypt; 5 Zhejiang University–Zhongyuan Institute, Zhengzhou, Henan, China; 6 Department of Biomedical Science, Kangwon National University 34962, Chuncheon, Gangwon, Republic of Korea; University of Illinois Urbana-Champaign, Urbana, Illinois, USA

**Keywords:** prophage, bacteriophage therapy, lysogeny, gut microbiota, dysbiosis, microbial diversity

## Abstract

Bacteriophages (phages), the dominant prokaryotic viruses that specifically target bacteria in the human gut microbiome, play a crucial role in maintaining intestinal balance, regulating bacterial populations, and preserving microbial diversity within the gut microbiota. While prophages can enhance bacterial virulence and antibiotic resistance, potentially posing health risks, they also provide beneficial functions, including enhancing host fitness, promoting immune modulation, and contributing to ecosystem resilience, which supports intestinal homeostasis. Human gut microbiota is essential for various physiological functions, including digestion, vitamin synthesis, immune modulation, and protection against pathogens. Dysbiosis, or microbial imbalance, is associated with various disorders such as inflammatory bowel disease, obesity, diabetes, and mental health disorders. Consequently, prophages are important considerations for developing therapies to prevent intestinal diseases. Recently, there has been significant interest in prophage induction in the gut due to its functional impacts on microbial dynamics, gut health, and disease modulation. Prophage induction can be regulated by diet, antibiotics, metabolites, gut health, lifestyle, and intestinal environments. However, compared with lytic phages, prophages remain underexplored, leaving gaps in our understanding of their functions within the gut. Therefore, further research is needed to fully elucidate the complex interactions between phages, prophages, and the gut microbiota, and their effects on health and disease. This knowledge could inform the development of phage-based therapies and improve therapeutic strategies for gut health.

## INTRODUCTION

The human gut microbiota is a complex ecosystem comprising trillions of microorganisms, including bacteria, viruses, fungi, and archaea, primarily residing in the gastrointestinal tract (1). Gut microbiota plays crucial roles in human health, including digestion, vitamin synthesis, immune regulation, and pathogen defense. Through complex intra-microbial interactions and host-microbe crosstalk, the gut microbiota dynamically modulates its composition, metabolic activities, and impacts systemic physiology (2). The dynamic composition and metabolic activities of gut microbiota significantly modulate host physiology through complex microbial-microbial and host-microbe interactions, influencing systemic homeostasis and health outcomes. Dysbiosis of intestinal microbiota during severe illness contributes to nosocomial infections, multi-organ failure, and associated negative clinical outcomes (3). This dysbiosis is associated with various disorders, including inflammatory bowel disease, obesity, diabetes, and mental health conditions (3). Therefore, understanding gut complex ecosystems is essential for developing targeted therapeutic strategies to maintain and restore gut health, potentially addressing a wide range of health conditions through microbiome modulation.

The human gut microbiota is predominantly composed of bacteriophages (phages) and bacteria, primarily from the phyla *Bacteroidetes* and *Firmicutes* (4, 5). Phages significantly influence microbial communities in the human gut that can regulate bacterial populations through density-dependent lysis, promoting microbial diversity, driving evolutionary changes, and stabilizing the overall microbial community (6). Most gut phages are temperate, existing as integrated prophages within bacterial genomes (4). These temperate phages can significantly impact the composition and function of the gut microbiome (7). However, the role of temperate phages in the gut environment remains less understood compared to lytic phages. The complex ecological role of phages arises from their extensive genetic diversity and dynamic interactions with bacterial hosts, presenting significant challenges in elucidating their impact on intestinal homeostasis and pathophysiology (6, 8). Particularly, prophages can modulate the equilibrium between commensal and opportunistic microbes in the gut microbiome, potentially contributing to gastrointestinal dysbiosis and associated health disorders. Hence, this review aims to comprehensively explore the intricate relationships between bacteria, phages, and prophages in shaping microbial diversity, microbial stability, and their potential connections to various gut disorders. In addition, this study focuses on addressing knowledge gaps in understanding various complex interactions and their implications for human health and disease.

## PHAGE AND PROPHAGE LIFECYCLE AND DIVERSITY IN THE HUMAN GUT

**Fig 1:**
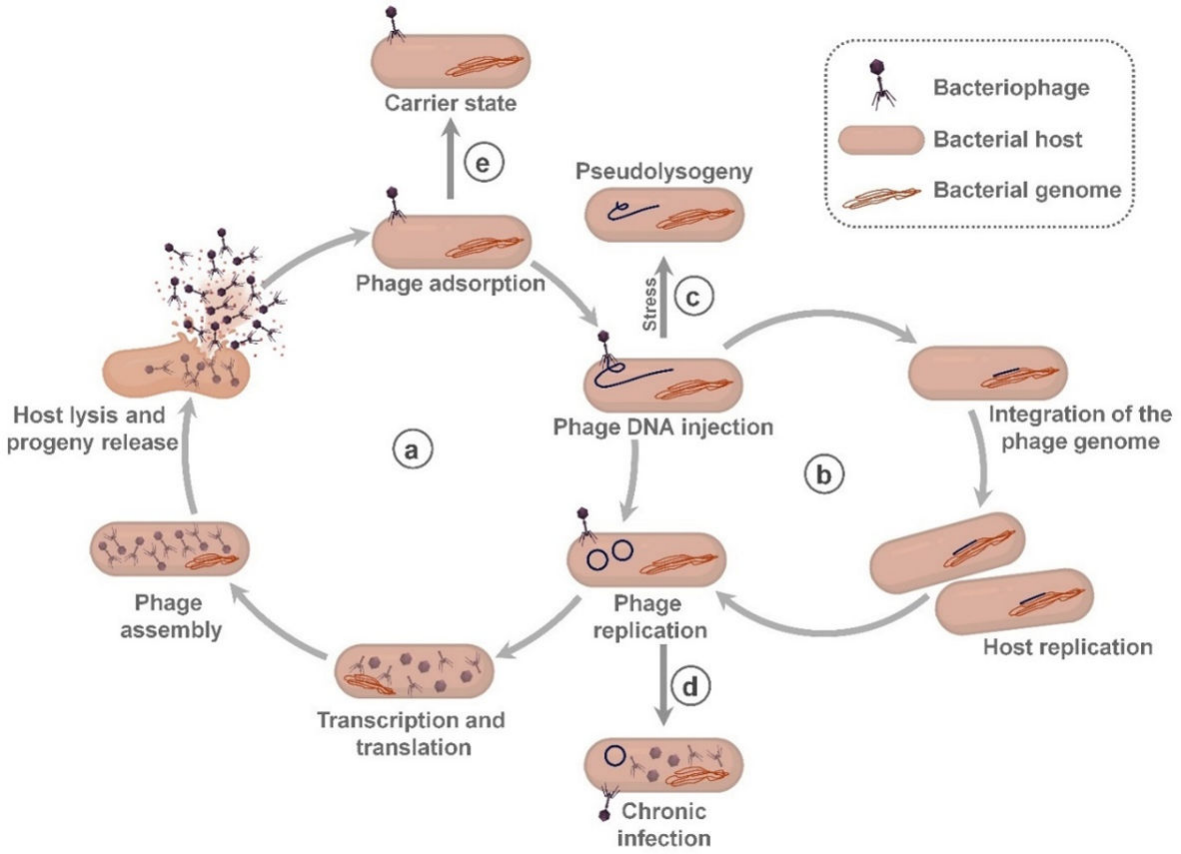
The schematic diagram illustrates distinct life cycles of phages (17). (**a**) Lytic cycle: Stages of viral replication leading to host cell lysis and release of new phage particles. (**b**) Lysogenic cycle: Phage genome integrates into the host genome, forming a prophage, and replicates along with the host genome. (**c**) Pseudolysogeny state: A transient state triggered by stress, where the prophage remains quiescent, and phage gene expression is downregulated, allowing host cell survival and division. (**d**) Chronic infection: Persistent phage infection where the host cell continues to grow and divide without cell lysis. (**e**) Carrier state: Phage persists as episomal DNA, distinguishing it from lysogeny; unlike chronic infection or pseudolysogeny, the genome is maintained extrachromosomally while the host survives.

Phages are the most abundant biological entities on Earth, thriving wherever their bacterial hosts exist, including the human gut microbiome (9, 10). In healthy individuals, phages potentially contribute to microbiome homeostasis due to their high host specificity and various life cycle states (Fig. 1). In the case of the lytic cycle, the phage ejects its genome into the host, where its genes are expressed and replicated to produce progeny, leading to cell lysis (11) (Fig. 1a). This process culminates in the release of progeny virions, perpetuating the infection cycle. In the lysogenic cycle (Fig. 1b), phage genomes can integrate as prophages with host genomes, which can significantly alter host bacterial genetics and physiology through lysogenic conversion and superinfection immunity. This process, known as a phage-driven regulatory switch, reprograms bacterial gene expression (12). Under nutrient stress, virulent phages may enter a pseudolysogenic state (Fig. 1c), failing to initiate the lytic cycle (13). Pseudolysogens harboring prophages often show reduced metabolic capacity (14). Chronic infection state (Fig. 1d) involves long-term phage replication within the host, with continuous progeny release without immediate cell lysis (15). The carrier state (Fig. 1e) exhibits a stable equilibrium between a host population and a phage infection. Unlike the lysogenic state, the phage genome exists in the extrachromosomal region and does not integrate into the host bacterial chromosome. Persistent production of lytic phages occurs in the presence of susceptible host cells, contributing to nutrient cycling and population stability in the gut ecosystem (16).

The prevalence and composition of the gut phageome in humans exhibit significant heterogeneity. For instance, the specific bacterial taxa, *Bifidobacterium* and their cognate phages, can be vertically transmitted from mother to infant via breastfeeding (18). CrAss-like phages have been vertically passed from mother to infants (19). The gut virome demonstrates a broader spectrum of phage classes, including *Caudoviricetes* and *Malgrandaviricetes* (formerly known as *Myoviridae*, *Podoviridae*, *Siphoviridae*, and *Microviridae*, with notable representation of tailed phages at the age of 2. Within *Caudoviricetes*, formerly categorized families such as *Myoviridae*, *Podoviridae*, and *Siphoviridae* exhibit distinct patterns of abundance. A decrease in *Podoviridae*-like phage abundance has been observed by the age of 3, whereas *Siphoviridae*-like phage prevalence remains relatively stable compared to earlier stages (20). The prevalence and abundance of virus-like particles, particularly tailed phages classified under *Caudoviricetes*, have been found to vary across different age groups. These fluctuations are influenced by environmental and nutritional factors (2). Phages belonging to *Malgrandaviricetes*, such as *Microviridae*, are detected throughout the gastrointestinal tract, including the terminal ileum, proximal colon, distal colon, and rectum, though typically in lower numbers (21). Specific phage taxa have been associated with human health outcomes (8). For instance, higher concentrations of *Caudoviricetes* members, including those previously referred to as *Siphoviridae*, have been associated with improved executive function and verbal memory (22). However, our understanding of phage diversity and dynamics in the human gut is limited and sometimes conflicting due to differences in experimental conditions, host populations, and detection methodologies (21). Reported prophage induction rates under antibiotics, dietary interventions, or other stressors vary widely across studies, highlighting uncertainties in current models of prophage activity and host interactions. The difficulty in culturing gut bacteria (>95%), including members of *Bacteroidaceae*, *Prevotellaceae*, *Ruminococcaceae*, and *Lachnospiraceae* families, hinders accurate representation of gut phage diversity in fecal samples (6). For example, *Bifidobacterium breve* and *B. longum* are known to harbor abundant prophages but remain resistant to lytic phage isolation, illustrating how host biology can complicate experimental validation and prophage-phage characterization (23). In addition, technical and methodological challenges complicate the interpretation of gut prophage data (24). For example, metagenomic binning often struggles to assign viral sequences to specific bacterial hosts, particularly in fragmented or low-abundance genomes, leading to underestimation or misclassification of prophage diversity (25). Viral particle enrichment techniques, such as filtration and density gradient centrifugation, may selectively capture or exclude certain phage types, biasing community composition analyses (26, 27). Furthermore, prophage prediction tools vary in sensitivity and specificity: some overpredict cryptic elements, while others fail to identify functional prophages, complicating the interpretation of prophage functionality and ecological roles (28). By explicitly acknowledging these methodological limitations, we provide a more critical perspective on conflicting findings and uncertainties in the gut phage literature. These considerations highlight general constraints in current studies, as reported by previous research, and underscore the importance of interpreting prophage data cautiously (29).

The presence of prophages within the human gut microbiome has been analyzed using a data set of 43,942 bacterial genomes representing 439 distinct species, leading to the identification of 105,613 putative prophage regions (30). Among these, 16,254 individual prophages represent a curated subset of complete or high-confidence prophages filtered for host specificity and sequence completeness, demonstrating a heterogeneous distribution across the gut bacterial community (30). Furthermore, results revealed that approximately 5.8% of identified prophages harbored toxin-encoding genes, while approximately 2.5% contained antibiotic resistance genes (30). Prophages carrying toxin-encoding genes can release toxins, potentially contributing to chronic diseases and negatively impacting gut health (31). Environmental factors within the gastrointestinal tract, including dietary composition, microbiome architecture, and host immune status, are implicated in the modulation of prophage induction. Specifically, dietary enrichment with vitamin B12 has been observed to augment *stx* prophage induction in Enterohemorrhagic *Escherichia coli* (32). Likewise, in lactic acid bacteria, exposure to short-chain fatty acids (SCFAs), fructose, and acetic acid has been demonstrated to elicit prophage induction (33).

The impact of age-associated phage dynamics on gastrointestinal and systemic health is likely complex and multifaceted. Across all age groups, non-stunted children exhibited a high relative abundance of Bacteroides and Firmicutes phages within their gut microbiota. In contrast, stunted children displayed a reduction in Bacteroides phages and an increase in Proteobacteria phages, particularly in older children (20). In younger individuals, a diverse and stable phage community may contribute to the establishment and maintenance of a healthy gut ecosystem (34). In contrast, alterations in phage composition, potentially characterized by increased prophage induction rates, may contribute to intestinal dysbiosis (34). Overall, the human gut microbiome harbors a diverse phage and prophage community that plays a crucial role in shaping microbial ecology and host health. Characterizing the diversity and function of prophages is essential for understanding their impact on human health and disease.

## ROLE OF PROPHAGES IN THE GUT MICROBIOTA

### Microbiome homeostasis and its regulatory mechanisms

The phage-bacteria relationship within the gut microbiome is a dynamic and intricate interplay. This complexity is driven by selective pressures that foster an evolutionary arms race between phages and their bacterial hosts (35). This gut ecosystem comprises active lytic phages, which reside extracellularly, and integrated prophages, which are dormant within bacterial genomes, due to unfavorable conditions. While lytic phages function as bacterial predators contributing to gut health by regulating pathogen proliferation and maintaining microbial balance, prophages can also confer beneficial effects, such as enhancing bacterial host fitness, facilitating adaptation, modulating immune responses, and contributing to ecosystem resilience, thereby supporting a stable and diverse microbiome through phage-bacteria interactions (36). Additionally, phage-bacteria interactions can alter the physiological and metabolic properties of gut bacteria, influencing the structure, function, and composition of the microbial community (9). These interactions may contribute to microbial diversity and individual-specific gut microbiota profiles.

Phages are key components and potential regulators of the gut microbiome, affecting various bacterial phyla, including *Bacteroidetes* and *Firmicutes,* through phage-host interactions (5). These interactions drive evolutionary changes (37) and regulate bacterial population density and distribution in the gut environment (38). The kill-the-winner concept (Table 1) describes how phages can reduce the population of predominant bacteria, preventing single-species dominance and maintaining diversity (2, 6, 39). In children, for example, the abundance and diversity of gut bacterial communities support more lytic phages and “kill-the-winner” dynamics. In contrast, the stable bacterial communities support the lysogenic cycle and “piggyback-the-winner” dynamics in adult (40). This regulation is crucial for balancing the gut microbiota and controlling bacterial concentrations (31). Phages contribute to bacterial strain diversity through lytic activity, supporting constant community dynamics. Moreover, the phages play a vital role in determining the composition, diversity, intestinal homeostasis, and function of gut microbiota.

**TABLE 1:** Overview of phage-bacteria interaction dynamics

Category	Dynamics	Description	Key feature	Reference
Co-evolution	Red Queen	Constant evolutionary adaptation between phages and bacteria	Diversification of both phage and bacterial populations	(41)
	Arms Race	Progressive development of defense and counter-defense mechanisms	Highly specialized interactions between phages and bacteria	(35)
	Cross-Resistance	Bacterial multi-phage resistance and phage’s broad-spectrum infectivity	Cyclical resistance and infectivity changes in the population	(42)
Population control	Kill-the-Winner	Abundant and dominant bacterial species are suppressed by phages	Maintenance of microbial diversity within the gut microbiome	(2, 6, 39)
	Leaky resistance	Partial resistance to phages in bacteria that reduces phage infectivity and continues survival	Coexistence between phages and bacteria at reduced efficiency	(43)
	Variable Selection	Periodic changes in the selective pressures on both phages and bacteria	Temporal variation in the dominance of phages and bacteria	(44)
Stable coexistence	Rock-Paper-Scissors	Bacterial strains with different resistance profiles and phages with varying infectivity	Cyclical interactions between phages and bacteria for continuous adaptation	(45)
	Trade-off	Trade-offs between different characteristics of phages and bacteria	Balance between phage predation and bacterial competition	(46, 47)
	Piggyback-the- Winner	Integration into the genomes of the most dominant bacteria as prophages	Enhanced stability of both bacterial and phage populations with mutual benefits	(6, 39)
Prophage-mediated	Lysogenic Conversion	Bacterial behavior with enhanced fitness in specific environments	Changes in bacterial population dynamics and interactions	(48)

In the gut environment, lytic phages with antimicrobial activity have the potential to shape microbial communities through selective pressure, while temperate phages enhance bacterial pathogenicity and coexistence (49). The lysogenic state is commonly observed in anaerobic bacteria, which are prevalent in environments such as the gut, deep sea, and deep soil (50). This state can confer potential benefits to phages when their bacterial hosts experience nutrient limitations or reside within biofilms (51). For example, fecal coliphages in healthy humans are typically low in abundance and predominantly temperate, often spontaneously induced from lysogenic bacterial hosts (2). Prophages are more prevalent in the gut than lytic phages, which play a significant role in microbial interactions, influencing community composition, bacterial stability, and bacterial diversity (31). Activation of prophages can sometimes provide ecological benefits by eliminating competing bacterial strains, facilitating horizontal gene transfer (HGT) of adaptive genes, or enhancing the host’s immune response against secondary infections (52).

Moreover, lysogenic conversion can alter bacterial phenotypes, potentially increasing virulence and antibiotic resistance, posing risks to human health (31). However, these same mechanisms can also provide advantages to the host microbiome, such as increased resilience to environmental stressors, maintenance of microbial balance, and promotion of beneficial host-microbe interactions. Both lytic and lysogenic phages regulate bacterial populations and promote microbiome stability; specific examples include Shiga toxin-encoding prophages in *E. coli*, fructose-induced prophages in *Lactobacillus reuteri*, and prophage-mediated lysis of *Faecalibacterium prausnitzii* (31, 53). In the gut, phages impact genetic diversity (54), bacterial resistance, and host immune responses (55). These processes increase bacterial diversity and drive the evolution of new traits in the gut microbiome.

### Prophage defense mechanisms in bacteria-phage interactions

Bacterial hosts have evolved various anti-phage defense mechanisms, including receptor modulation, restriction-modification systems, clustered regularly interspaced short palindromic repeats (CRISPR)-associated protein, abortive infection, toxin-antitoxin systems, and superinfection exclusion (31, 56). In addition to these well-established systems, several recently characterized defense mechanisms, such as the cyclic oligonucleotide-based antiphage signaling system (57, 58), the defense island system associated with restriction-modification (59), Zorya (60), Gabija (61), and Shedu (62), have further expanded our understanding of bacterial immunity against phages. To overcome bacterial defense systems, prophages have the potential to control antiphage defense systems and the phage lysogenic cycle (63). Prophages, as the integrated form of lysogenic phages, exert a more significant influence on their bacterial hosts, establishing a reciprocal relationship (19). Prophage induction can impact bacterial genetic diversity by facilitating HGT in various pathways. It has gained attention for its potential to modulate bacterial fitness by transferring beneficial genes (64), microbial community structure (65), and enhance host immunity against secondary phage infection (64).

The gut phageome can contribute to enhancing the host immune system by targeting and inhibiting pathogenic bacteria, thereby supporting a healthy gut microbiome. In addition, phages interact with the host immune system, influencing immune responses and potentially impacting the composition of the gut microbiome. Phages can influence innate immune responses by affecting phagocytosis and cytokine production, while also impacting adaptive immunity by altering antibody production (55). Induced phage, for example, causes the overexpression of IL-10, the suppressor of cytokine signaling 3, and IL-1 receptor antagonist, to reduce inflammation. Additionally, it can suppress T-cell activation during phage-host infection and downregulate pro-inflammatory cytokines (IL-1β, NF-κB), chemokines, cytokines (TNF-α, IL-6, and IL-8), and Toll-like receptor 4 (66, 67). Moreover, phages provide a novel therapeutic strategy by connecting their ability to suppress inflammation through pathogen clearance, cytokine modulation, and oxidative stress reduction, thereby promoting improved human health, enhanced immune function, and accelerated recovery (68). The gut phageome plays crucial roles in shaping the microbiome, influencing host health, and maintaining gastrointestinal fitness. Moreover, phages can inhibit pathogens and support beneficial bacteria, contributing to a balanced microbial community.

## MECHANISM OF PROPHAGE INDUCTION

**Fig 2:**
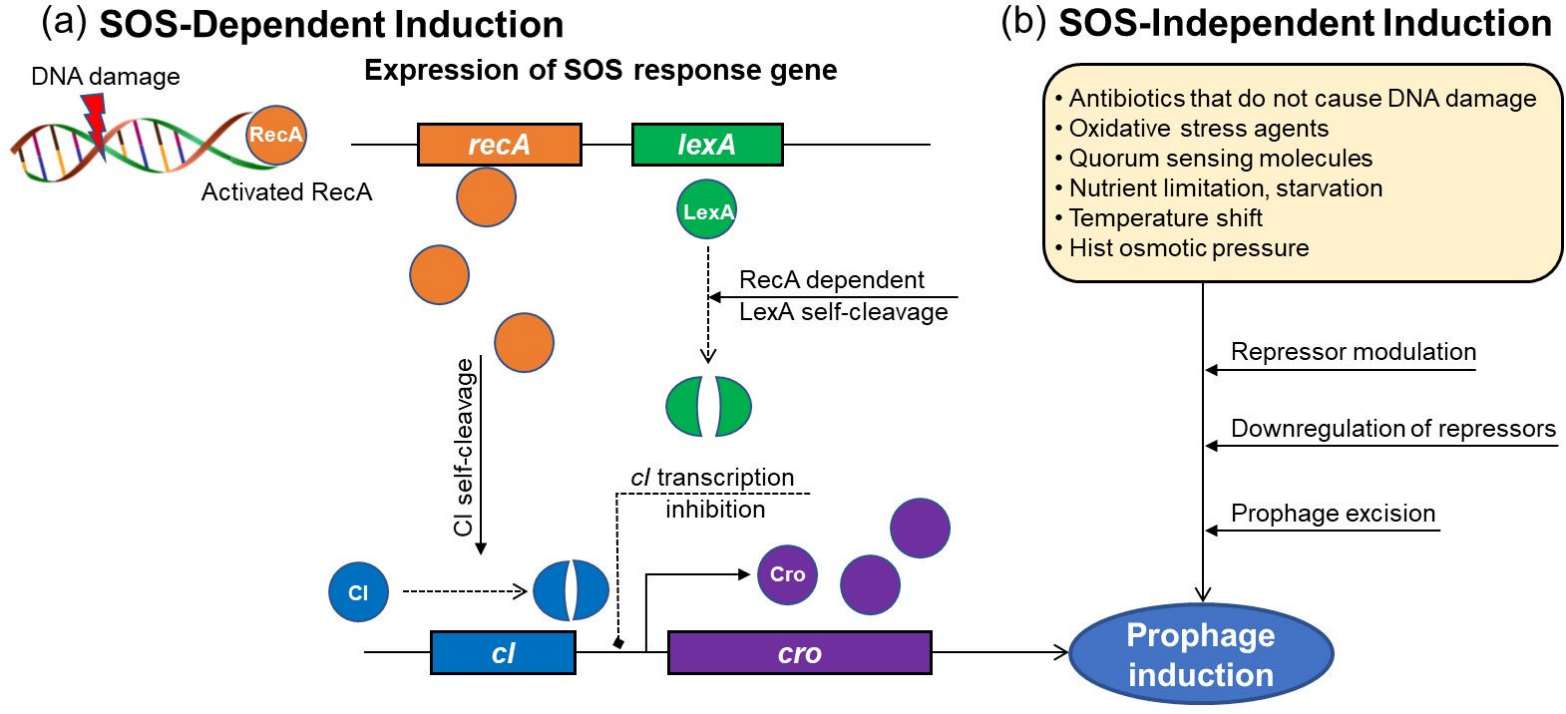
SOS-dependent and SOS-independent prophage induction. DNA-damaging agents trigger the SOS-dependent pathway (**a**) by activating RecA, which promotes cleavage of LexA and the phage cI repressor. The SOS-independent pathway (**b**) occurs without RecA and is triggered by stressors such as nutrient limitation, cell wall stress, quorum signals, or temperature shifts, often via phage-encoded anti-repressors.

Prophages can be induced by both SOS-dependent (DNA damage response–mediated) and SOS-independent pathways to enter the lytic cycle. These mechanisms play a crucial role in shaping the gut microbiome by regulating bacterial populations and influencing the dissemination of genetic material. Prophage induction is generally stimulated by the bacterial SOS response, which coordinates cellular reactions to DNA damage through interactions between the RecA protein and the LexA repressor (Fig. 2). The RecA protein acts as a signal for prophage induction. RecA-ssDNA filaments promote self-cleavage of LexA repressor in response to DNA damage, enabling the expression of SOS genes that facilitate DNA repair (31) (Fig. 2a). Prophage induction is a cascade process from the SOS response to RecA activation and *c*I repressor cleavage, which leads to transcription of virulence gene stx in the foodborne pathogen *E. coli* O157:H7 (69). The *c*I repressor binds to operators on phage DNA to maintain the lysogenic state and prevent lytic gene expression. In contrast, anti-repressor proteins can regulate the switch to the lytic cycle as a survival strategy under stress conditions, causing DNA damage (70, 71). Moreover, anti-repressor proteins inhibit repressor proteins, while repressor proteins and regulatory sequences within the phage genome collectively control the crucial shift from the lysogenic to lytic cycle (70, 71). The *c*I repressor of λ phage, for example, binds to the promoter of lytic cycle genes, preventing their transcription and suppressing the lytic cycle. This allows the phage to enter a lysogenic state, integrating its genome into the host chromosome. Recombination events driven by host or phage-encoded recombinases can cause genetic diversity in phage genomes, leading to the emergence of new variants with altered properties (72). Inflammatory factors such as reactive nitrogen species (RNS) and reactive oxygen species (ROS) can stimulate the SOS response, potentially increasing phage anti-repressor expression (73). This mechanism enhances the phage’s ability to evade host defenses and initiate an effective infection.

The interaction between RecA and LexA can change due to the complex intestinal environment. In the gut, RecA is involved in numerous DNA repair processes beyond SOS induction (74). The multiple DNA damage-inducing agents, such as ROS and bacterial toxins, could lead to competition for RecA binding, potentially delaying or reducing the availability of RecA for LexA cleavage (75). Other bacterial proteins or metabolites could potentially interact with LexA, altering its binding affinity for operator regions or its susceptibility to RecA-mediated cleavage (76, 77). Inter-bacterial signaling molecules, such as quorum-sensing molecules or antimicrobial peptides, could indirectly influence prophage induction by altering the overall stress response of the host bacterium (78, 79). These signals may induce or suppress other DNA repair systems, thereby affecting the availability of RecA. The intestinal environment is subject to fluctuations in pH, nutrient availability, and redox potential. These fluctuations can induce various stress responses in bacteria, some of which may overlap or interfere with the SOS response. Therefore, while the RecA-LexA interaction is crucial, it is likely subject to context-dependent modulation in the intestine. RecA-LexA dynamics in the gut are context-dependent: competing DNA-repair processes and environmental stresses such as ROS, toxins, and quorum signals can delay or modify SOS-mediated prophage induction (74, 76, 78–80).

In addition, prophage induction can be triggered by SOS-independent mechanisms (Fig. 2b). SOS-independent prophage induction can be mediated by various mechanisms such as quorum sensing, cellular stress responses (e.g., heat shock, oxidative stress), environmental clues (e.g., nutrient limitation, pH, UV), and bacterial metabolic pathways, which can trigger prophage excision and activation. Prophage Pf4, integrated into the *Pseudomonas aeruginosa* PAO1 genome, was activated in a *mvaT mvaU* double mutant but not in single mutants, indicating a synergistic role of *mvaT* and *mvaU* in repressing prophage induction (81). Similarly, inhibition of the transcription termination factor Rho can regulate prophage maintenance in *E. coli* (82). In addition, quorum sensing potentially influences prophage induction by affecting ecological networks within bacterial communities in the gut. Tan et al. (83) strengthen the piggyback-the-winner model by revealing that prophage induction in *Vibrio anguillarum* is suppressed due to the quorum-sensing activation in the host cells. This study suggests that quorum sensing may act as a regulatory mechanism that is associated with bacterial density to prophage induction rates. Intestinal components, such as bile acids, and bacterial metabolites, like SCFAs, can induce prophages in *Salmonella enterica* (84) and *Lactobacillus* species, such as *L. reuteri* ATCC 55730, *L. lactis* NZ9000, and *L. reuteri* 6475, respectively (85), through alteration in the gut microbial community and metabolic activity. These diverse induction mechanisms highlight the complex interplay between prophages, their bacterial hosts, and the gut environment, significantly enhancing phage-bacteria interactions within the gut ecosystem.

## PROPHAGE INDUCTION IN THE GUT

**Fig 3:**
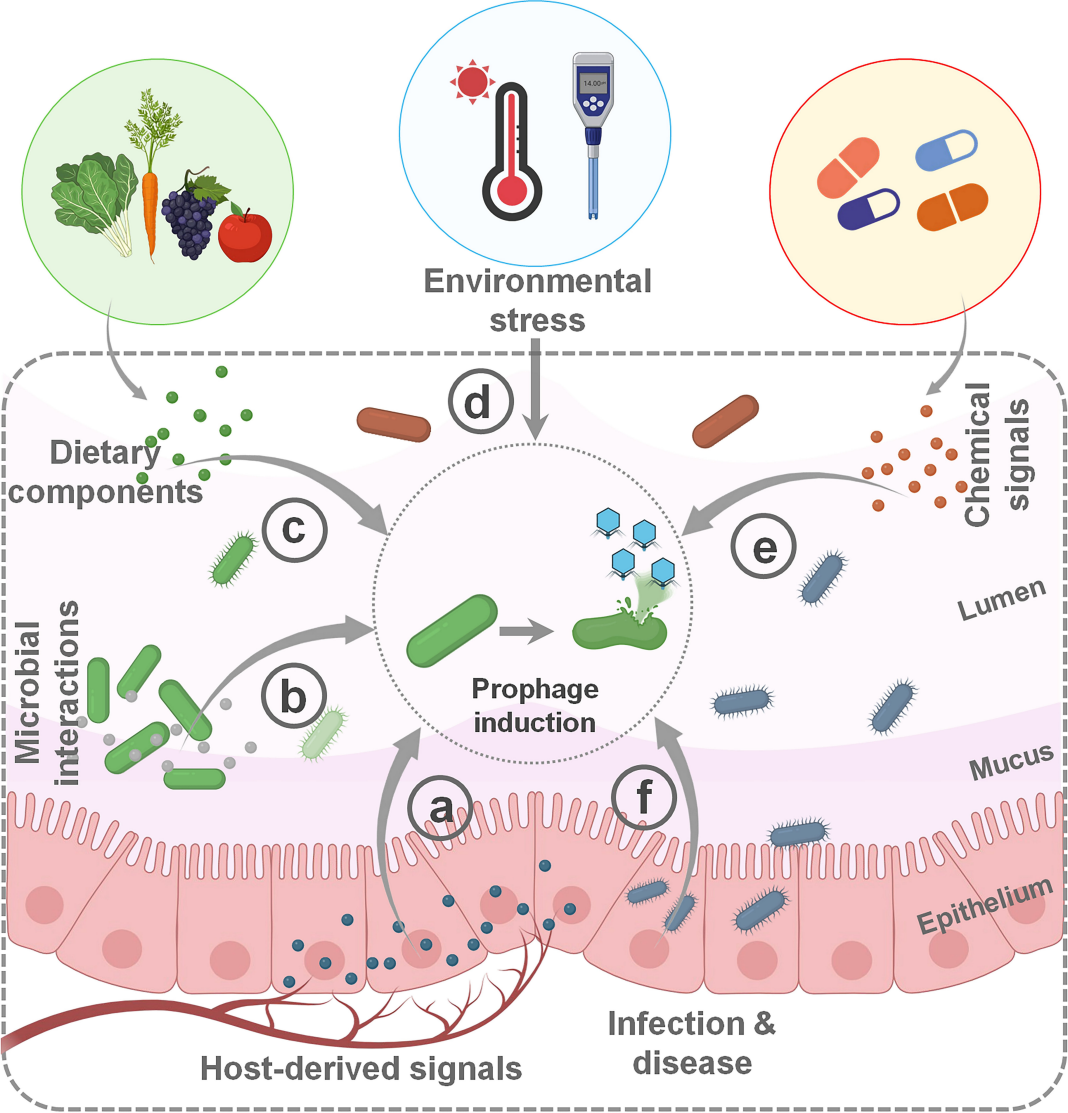
Factors triggering prophage induction in the gut microbiota. (**a**) Host-derived signals. (**b**) Microbial interactions. (**c**) Dietary components. (**d**) Environmental stress. (**e**) Chemical signals. (**f**) Infection and disease.

Prophage induction in the gut microbiota is triggered by various factors (Table 2), including host signals such as immune responses and hormones (Fig. 3a), microbial interactions such as competition and quorum sensing (Fig. 3b), dietary components including fibers, sweeteners, and polyphenols (Fig. 3c), environmental stressors like nutrient limitation, osmotic changes, and pH shifts (Fig. 3d), chemical signals such as antibiotics, bile acids, and antimicrobial peptides (Fig. 3e), and infection or inflammation (Fig. 3f) (53). These triggers can promote HGT of virulence and resistance genes, reshaping both bacterial and phage populations. For instance, carbadox can induce Shiga toxin (*Stx*)-encoding prophages in *E. coli* strains (86). Different classes of antibiotics, including β-lactams and quinolones, have been shown to induce prophages in *Staphylococcus aureus* (87) and Shiga toxin-producing *E. coli* (88), often by triggering the bacterial SOS response (Fig. 3e). Environmental toxins like benzo[*a*]pyrene-diol-epoxide can also induce multiple prophages that potentially disrupt beneficial gut bacteria *Lactobacilli*, which can disrupt community composition (89) (Fig. 3d). Natural compounds, such as cinnamon oil (CO), can modulate prophage induction. CO has been shown to influence gene expression in *E. coli* O157:H7, potentially reducing *Stx* production by suppressing key mediators of the SOS response and quorum sensing. Specifically, CO inhibits RecA, polynucleotide phosphorylase (PNPase), and poly(A) polymerase (PAP I), leading to decreased *Stx* production. Additionally, CO suppresses the expression of *qseBC* and *luxS* genes, which are involved in universal quorum-sensing signaling (69) (Fig. 3b). Furthermore, CO upregulates the expression of oxidative stress-related genes (*oxyR*, *soxR*, and *rpoS*), resulting in delayed bacterial growth and reduced induction of Stx2 prophages in *E. coli* O157:H7 (69).

**TABLE 2:** Prophage-inducing agents and mechanisms

Prophage inducer	Source	Prophage (host)	Mechanism	Reference
Bile salts	Biological detergents	P22 (*Salmonella* Typhimurium), CJIE1 and CJIE4 (*Campylobacter jejuni*)	DNA damage^*a*^, phage repressor downregulation^*a*^	(90)
CRISPR-interference	Bacterial system	Lambda and P2 (*E*. *coli*)	Prophage repressor inactivation^*a*^	(91, 92)
Gamma irradiation	Food processing technology	CP-933V and BP-933W (*E. coli* O157:H7)	ROS production-mediated oxidative stress^*a*^	(93)
ROS, RNS, and hypochlorite	Reactive intermediates in inflammation	SopEΦ (*S*. Typhimurium)	Inflammatory stresses^*a*^	(73)
Peptides	Signaling molecules	phi3T (*Bacillus subtilis* BEST7003)	Cell density-dependent signal decay	(94)
Acyl-homoserine-lactone autoinducers (AHL)	Quorum-sensing molecules	ϕH20 (*Vibrio anguillarum*)	Cell density-dependent regulatory system	(83)
Furanosyl borate diester	Quorum-sensing molecules	T1 (*E*. *coli*)	Prophage induction regulator- (Pir-) dependent activation	(79)
3,5-Dimethyl-2-pyrazinol	Quorum-sensing molecules	VP882 (*V*. *cholerae*)	Phage antirepressor activation (Qtip mechanism)	(78)
Fructose	Dietary component	LRɸ1 (*L. reuteri* 6475)	Acetate accumulation^*a*^	(95)
SCFAs	Dietary component	LRɸ1 and LRɸ2 (*L. reuteri* 6475)	Acetate accumulation^*a*^	(95)
Fats	Dietary component	Firmicutes-associated prophages	Gut dysbiosis	(31)
Xylose	Food additive	PBSX (*B*. *subtilis*)	PL promoter induction (spore germination)	(96)
β-lactams	Antibiotic	φ80α and φ11 (*S*. *aureus*)	RecA/LexA activation^*a*^	(87)
Ciprofloxacin	Antibiotic	φ11 and 80α (*S*. *aureus*)	DNA gyrase inhibition^*a*^	(97)
Heat shock	Thermal stress	Lambda (*E. coli* K-12)	CII activity downregulation^*a*^	(98)
Acid exposure	Acid stress	phiHP33 (*Helicobacter pylori*)	DNA damage^*a*^	(99)
Lactococcin 972 (Lcn972)	Bacteriocin	ΦLC3 (*L. lactis* IMN-C1814)	DNA damage^*a*^	(100)
Colibactin	Bacterial metabolites (genotoxin)	Lambda (*E*. *coli* BW25113) and Phi11 (*S*. *aureus*)	RecA activation^*a*^	(101)
Colicin	Colicinogenic bacterial metabolite	Lambdoid prophage (*E*. *coli*)	RecA activation^*a*^	(102)
Pyocyanin	*Pseudomonas* metabolite	phiMBL3 (*S. aureus*)	ROS production (intracellular oxidation level)	(103)

^*a*^ Indicates bacterial SOS responses.

The mammalian gut harbors a complex microbial community, including bacteria-carrying prophages. The balance of gut bacteria is influenced by both dietary (bottom-up) and regulatory (top-down) mechanisms. These prophages can be induced by various triggers, including foods and dietary factors such as fructose intake and fiber consumption (Fig. 3c) (34, 104). Different diets can alter the levels of gut bacteria by inducing prophages, suggesting a new approach to shaping the human gut microbiome (104). For example, Oh et al. (85) demonstrated that a fructose-enriched diet can activate prophages in *L. reuteri* and enhance phage production. This effect is mediated by the activation of the Ack pathway, which generates acetic acid and triggers a bacterial stress response. Mutants of *L. reuteri* that lack the Ack pathway or the stress response protein RecA exhibit low phage production. A study demonstrates that diet and dietary metabolites directly induce prophages in gut symbionts, linking dietary, antimicrobial, and chemical triggers as common induction pathways (85) (Fig. 3e). Dietary changes, such as adopting a gluten-free diet, can influence the abundance of specific gut phage groups, including crAss-like phages, members of the *Microviridae* family (ssDNA phages), and tailed dsDNA phages such as formerly *Podoviridae*. Additionally, *Microviridae* are more prevalent in high-fat diets, while *Caudovirales* are more abundant in normal diets (105). Different dietary patterns are associated with distinct phage profiles. For instance, high-fat diets are linked to an increased prevalence of lysogenic phages, whereas low-fat diets are associated with a higher prevalence of lytic phages (7). Prophage induction in the intestine has drawn significant interest due to its potential functions in modulating microbial community structure and influencing host immunity (Fig. 3f).

Sutcliffe et al. (52) investigated prophage induction in human gut bacterial communities under real-life conditions, like medications. This study demonstrated that a range of orally administered pharmaceuticals, including chemotherapeutic agents (fludarabine and busulfan), non-steroidal anti-inflammatory drugs (ibuprofen, diclofenac, and tolmetin), cardiac medications (digoxin), antibiotics (norfloxacin, ciprofloxacin, mitomycin C, ampicillin, and streptonigrin), and temperate analgesics (acetaminophen), exhibited inhibitory effects on bacterial growth potentially through interference with DNA replication and prophage induction (55%). Furthermore, the study revealed species-specific prophage induction within *Bacteroides caccae* and *Clostridium beijerinckii* (52). In addition, studies on *B. breve* and *B. longum* have shown that mitomycin C treatment can trigger prophage excision, replication, and lysis, highlighting concrete mechanisms by which prophages contribute to bacterial adaptation and shaping of gut microbial communities (23).

## PHAGEOME DYNAMICS IN GUT ECOLOGY AND EVOLUTION

### Bacterial fitness and community dynamics

The gut phageome can significantly influence bacterial fitness and community structure (106). Temperate phages can either lyse the host cell or enter a lysogenic state, can transfer virulence genes from one host to another through lysogenic conversion, potentially increasing the host’s fitness or enabling it to acquire pathogenic traits, which could facilitate colonization of new niches (107). The decision to lyse or lysogenize is influenced by factors like bacterial cell state, phage quantity, and environmental conditions. This decision represents an evolutionary strategy for temperate phages to maximize bacterial fitness (108). Prophages integrated into the host genome often provide selective advantages to the lysogenic state over non-lysogens. Bondy-Denomy and Davidson (106) highlight the multifaceted role of prophages in bacterial fitness and adaptability through various mechanisms, including coinfection exclusion, superinfection immunity, increased genetic diversity, stress resistance, biofilm formation, virulence factor production, competitive nutrient acquisition, population control, and HGT.

Additionally, the impact of prophage on bacterial fitness can vary depending on the specific genes encoded by the prophage and environmental conditions. Prophages can enhance bacterial fitness through various mechanisms, including facilitating genome rearrangements, disrupting non-essential genes, providing immunity to related phages, killing competing bacteria, introducing new traits, and potentially silencing host genes (14, 109). These fitness factors can be categorized into three classes, including survival factors (e.g., nutrient uptake systems), defense factors (e.g., antigen masking), and offensive factors (e.g., toxins), which contribute to bacterial adaptability and survival in diverse environments (109). This intricate interplay between temperate phages and their bacterial hosts plays a crucial role in shaping the gut microbial ecology. Understanding these complex interactions is essential for comprehending the impact of phages on human health and disease.

### Evolutionary roles in gut environments

Prophages play a significant role in shaping the gut ecosystem, influencing nutrient cycling by regulating bacterial populations and their metabolic activities. Furthermore, prophages drive evolutionary processes by introducing genetic diversity through HGT and enabling bacteria to adapt to various environmental conditions (110). The prevalence of active lysogens and prophages suggests extensive lysogenic infections, influencing host gene expression by interrupting functional and regulatory genes through phage integration and excision events (12). The coevolution between phages and bacteria involves a continuous cycle of defense and counter-defense to maintain competitive and adaptive advantages, known as the Red Queen hypothesis (41). López-Beltrán et al. present a study that demonstrates the hypothesis that the evolutionary arms race between prokaryotic hosts and mobile genetic elements is a major force shaping microbial community ecology and evolution (111). Long-tailed phages may possess an evolutionary advantage in the intestinal environment due to their rotational movement, which can enhance attachment to host cells and facilitate efficient infection (112). Lysogenic phages often do not compete with dominant species but exploit their success, a dynamic known as piggyback-the-winner (Table 1) (6, 39). This approach allows them to persist by associating with successful bacterial populations. Certain bacterial phyla, such as *Firmicutes* and *Proteobacteria*, are more frequently lysogenized by temperate phages compared to *Bacteroidetes* and *Actinobacteria* (7).

## IMPACT OF PHAGEOME ALTERATIONS ON GUT HEALTH

Diseases like *Clostridioides difficile* infections, norovirus-associated diarrhea, ulcerative colitis, and Crohn’s disease can alter gut microbiota. These diseases can cause intestinal dysbiosis, which is an alteration in the composition of the gut microbiota and alters phageome structure (34). Phage levels in the gut vary among individuals with different physiological states. For example, healthy individuals typically have lower phage abundance compared to patients with increasing clinical symptoms who often harbor a majority of virulent phages (2). In some cases, significant changes in gut microbial composition are associated with intestinal disorders, including decreases in beneficial commensal bacteria like *F. prausnitzii* and *Bifidobacterium* species, and increases in pathogenic *Enterobacteriaceae* and *Bacteroides* species. Virulent and free-floating phages are prevalent among individuals with intestinal diseases due to the overuse of antibiotics. Additionally, exogenous phages can induce biofilm formation, potentially leading to severe diseases, even without direct bacterial infection. For instance, Lourenco et al. (2022) (113) found that *E. coli* cells lacking either the *fliA* or *rfaL* gene exhibited significantly increased biofilm formation in the presence of phage CLB_P3, suggesting a potential bacterial defense mechanism against phage infection.

Prophage-inducing mechanisms can significantly impact microbiota stability and composition, leading to both beneficial and harmful consequences. By understanding and manipulating prophage activation, researchers have developed therapeutic strategies to treat deadly bacterial diseases, such as Shiga toxin-producing *E. coli* infection (31). Meessen-Pinard et al. (114) demonstrated a significant increase in phage ФMMP04, up to 9 log PFU/mL, due to prophage induction in the presence of quinolones in both *in vitro* and *in vivo* models of *Clostridium difficile* infection. The previous study concluded that prophage induction may contribute to both eubiosis and dysbiosis by altering the microbial community structure.

Dysbiosis has been linked to various pathological conditions, including diabetes, inflammatory bowel disease, Crohn’s disease, ulcerative colitis, irritable bowel syndrome, obesity, liver cirrhosis, rheumatic syndromes, metabolic syndrome, neurological disorders, and various gastrointestinal cancers, such as colorectal, esophageal, hepatocellular, gastric, and pancreatic cancer. As an example, colorectal cancer patients exhibit increased bacterial diversity and decreased temporal stability in their gut microbiome compared to healthy individuals, leading to high *Bacteroides* levels and low *Firmicutes* levels (115). To improve gut health, the gut phageome plays a fundamental role by modulating microbial communities and influencing immune responses (116). The abundance of phages, which constitute 17% of the human fecal metagenome, is recognized as a potential modulator of the gut ecosystem (10, 117). More specifically, prophages with beneficial properties, such as superinfection immunity and coinfection exclusion, can lead to alterations in the gut microbial community.

Barr et al. (118) proposed a model where phages can adhere to mucus layers, providing a non-host-derived antimicrobial defense. This defense mechanism works by preventing bacterial colonization of mucosal surfaces. Moreover, the Kill-the-winner dynamic interaction between phages and metazoan hosts represents a unique form of symbiosis. Similarly, free phages adsorbed onto bacteria in the gut can activate macrophages in mouse models, leading to reduced inflammation and cytotoxic damage (119). Overall, phages are highly associated with gut health, and their genomes hold the potential for preventing, understanding, and treating gut diseases.

## DIAGNOSTIC AND THERAPEUTIC STRATEGIES

**Fig 4:**
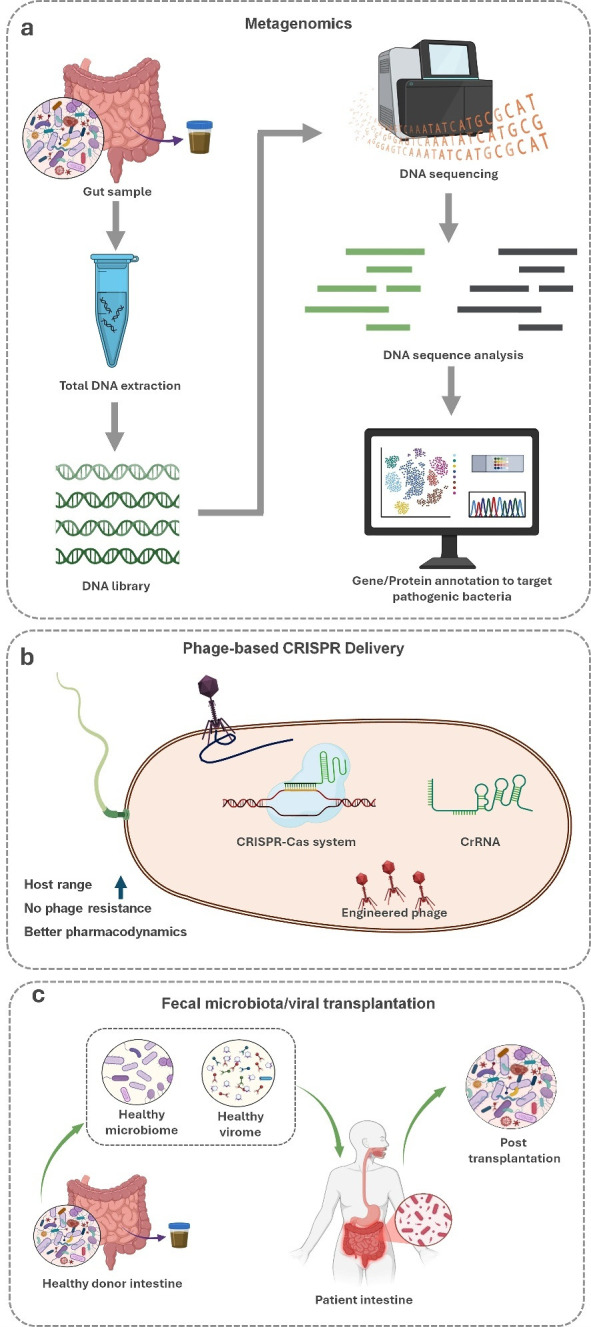
Therapeutic and diagnostic approaches for modulating gut microbiota. (**a**) Virulent or toxic genes of pathogens can be identified through metagenomic sequencing. (**b**) CRISPR-Cas systems can be employed to engineer phages for targeted therapy. (**c**) FMT and FVT involve the transplantation of fecal microbiota or viral communities to restore a healthy gut ecosystem.

Advanced approaches such as metagenomics, CRISPR-Cas, fecal microbiota transplantation (FMT), and fecal viral transplantation (FVT) have been developed for diagnosing gut pathogens and modulating the gut microbial community through phage-based strategies (Fig. 4). However, the application of phage therapy in intestinal diseases remains challenging due to several factors influencing its unpredictability. These factors include host-specificity of phages, pre-existing immune clearance of phages by the host, bacterial heteroresistance, genetic mutations in target bacteria, and variability of the gut environment, such as pH, nutrient availability, and microbial community composition (37, 120). Metagenomic sequencing offers a comprehensive approach for characterizing microbial communities, enabling the detection of both known and novel pathogens directly from stool samples without prior culturing (121) (Fig. 4a).

Recent advancements have highlighted the potential of lytic phages for phage therapy, which can prevent bacterial virulence resulting from lysogenic conversion (31). Phage therapy can restore gut balance either through lytic phages that eliminate pathogenic bacteria or through controlled prophage induction that reshapes microbial composition. Clinical case reports demonstrate that pre-existing immunity against phages or spontaneous bacterial mutations can disrupt therapeutic efficacy, highlighting the need for personalized phage therapy strategies (122). Strategies to optimize therapy include regulating bacterial SOS responses, controlling prophage-inducing signals, managing intestinal inflammation, and careful antibiotic use (31). Collectively, these strategies position prophage induction as a multidimensional therapeutic approach that can both suppress pathogens and support microbiome homeostasis. For example, Cornuault et al. (123) demonstrated that prophage induction led to the lysis of a key commensal bacterium, *F. prausnitzii*, in a mouse model of inflammatory bowel disease.

Leveraging phages as delivery vehicles for CRISPR/Cas9 systems represents an innovative therapeutic approach, enabling targeted genetic modifications within the gut microbiome (124) (Fig. 4b). The effectiveness of this strategy depends on precise phage selection to avoid off-target effects, highlighting the need for optimized delivery mechanisms and specificity (125). Similarly, FMT involves transferring stool from a healthy donor to a recipient to restore gut microbiota balance (Fig. 4c). For example, *Clostridium difficile*-associated disease may cause dehydration, sepsis, diarrhea, and death due to the production of toxins in the gut. To restore the natural microbiome, FMT has been employed to effectively treat *Clostridium difficile* infections and potentially cure the disease (121). FVT provides additional advantages, including minimizing the risk of transferring pathogenic bacteria and selectively delivering therapeutic phages (126). Overall, personalized phage therapy, considering host immunity, gut environment, and bacterial heterogeneity, is essential to maximize efficacy and minimize unpredictability, underscoring the need for patient-specific phage selection and monitoring.

Prophage research further provides insights into gut microbial ecology, with potential applications as biomarkers for metabolic and gut diseases. Since temperate phages integrate into host genomes, metagenomic recovery of individual bacterial genomes enables the study of lysogeny in natural communities, including the gut microbiome (127). Quantifying phages is critical for understanding their roles, as prophages can constitute 20%–50% of free phages and actively shape gut microbiota through prophage induction (37). Current studies focus on modulating gut microbial communities for therapeutic purposes. Understanding individual phage profiles is crucial for developing personalized microbiome-based therapies. The therapeutic potential of prophages lies in their ability to influence microbial balance and gut health through mechanisms such as controlled induction and genetic regulation. By integrating CRISPR/Cas systems, these approaches can selectively modify gut bacterial genomes to influence functions and interactions. In this context, regulating bacterial lysogenic conversion is essential for preventing disease and developing effective therapeutic strategies (31). Prophage research provides a valuable framework for improving pathogen control and gut microbiome regulation.

## CONCLUSIONS AND FUTURE DIRECTIONS

The gut phageome is a vital component of the gut microbiota with significant implications for human health and disease. Advanced technologies such as phage metagenomics and FVT have greatly contributed to our understanding of this complex system. Phage metagenomics allows for detailed genomic analysis of viral communities in the gut, while FVT involves transferring viral communities from donor feces to recipients to alter gut microbiota compositions. Although these approaches have expanded our knowledge, substantial challenges remain in fully characterizing and functionally interpreting the gut phageome. Culturing bacteria and understanding the diversity of gut phages continue to be difficult tasks. Advances in sequencing technologies have provided deeper insights into phage diversity and function. However, the complete scope of phage diversity and its implications for health are still not fully understood. Most research on prophages has focused on a limited number of bacterial species, resulting in an incomplete picture of the prophage diversity in the human gut. Moreover, the dynamics of prophage induction and lysis are not fully understood. Many viral populations remain unclassified, often referred to as “viral dark matter.” Metagenomic and computational approaches are increasingly helping to address this challenge by enabling the assembly of novel viral genomes, the prediction of phage-host interactions, and the functional annotation of uncharacterized viral sequences. Phage stability is another critical factor in phage therapy, as phages can vary in species and formulations. Additionally, phages can mutate or lose infectivity during extended storage periods. Furthermore, there is a lack of standardized protocols for the quality and safety of phage preparations. These limitations highlight the need for further research to better understand the roles of phages in shaping gut microbial communities and their impact on host health. Future research should address several unanswered questions: How do prophage induction rates vary across individuals and environmental conditions, and how do they influence microbial stability or dysbiosis? What mechanisms govern the balance between lysogeny, pseudolysogeny, and chronic infection in the gut? How do dietary factors, antibiotics, and host immunity shape prophage activity? To what extent do phages contribute to HGT and antibiotic resistance spread in the gut microbiome? What is the role of archaeal viruses in shaping gut ecology, and how might they influence human health? In addition, can phage-host interaction networks be modeled to predict therapeutic outcomes, and how can we harness this knowledge to design safe, personalized phage-based interventions? Advances in metagenomics and computational tools will aid in uncovering the complex interactions between phages and the gut microbiome. This is crucial for bridging the gap in understanding phage interactions within gut microbiota, which could pave the way for innovative treatments for gut-related diseases. Finally, future research should also expand to include the considerable diversity of archaeal viruses in the gut. There is a clear need to investigate these components of the virome, as preliminary evidence suggests they hold significant, though still uncharacterized, ecological roles and influence on host interactions.
